# Clinic-epidemiological evaluation of ulcers in patients with leprosy sequelae and the effect of low level laser therapy on wound healing: a randomized clinical trial

**DOI:** 10.1186/1471-2334-10-237

**Published:** 2010-08-10

**Authors:** Josafá G Barreto, Claudio G Salgado

**Affiliations:** 1 Dermato-Immunology Laboratory UEPA/UFPA/Marcello Candia, Marituba, Pará, Brazil; 2 University Campus of Castanhal - UFPA - Castanhal, Pará, Brazil; 3 Institute of Biological Sciences - UFPA, Belém, Pará, Brazil

## Abstract

**Background:**

*Mycobacterium leprae *is the only pathogenic bacteria able to infect peripheral nerves. Neural impairment results in a set of sensitive, motor and autonomic disturbances, with ulcers originating primarily on the hands and feet. The study objectives were to analyze the clinic-epidemiological characteristics of patients attended at one specialized dressing service from a leprosy-endemic region of the Brazilian Amazon and to evaluate the effect of low level laser therapy (LLLT) on wound healing of these patients.

**Methods:**

Clinic-epidemiological evaluation of patients with leprosy sequelae was performed at the reference unit in sanitary dermatology of the state of Pará in Brazil. We conducted anamnesis, identification of the regions affected by the lesions and measurement of ulcer depth and surface area. After that, we performed a randomized clinical trial. Fifty-one patients with ulcers related to leprosy were evaluated, twenty-five of them were randomly assigned to a low level laser therapy group or a control group. Patients were treated 3 times per week for 12 weeks. Outcome measures were ulcer surface area, ulcer depth and the pressure ulcer scale for healing score (PUSH).

**Results:**

Ninety-seven ulcers were identified, with a mean (SD) duration of 97.6 (111.7) months, surface area of 7.3 (11.5) cm^2^, and depth of 6.0 (6.2) mm. Statistical analysis of the data determined that there were no significant differences in the variables analyzed before and after treatment with low level laser therapy.

**Conclusions:**

Ulcers in patients with leprosy remain a major source of economic and social losses, even many years after they have been cured of *M. leprae *infection. Our results indicate that it is necessary to develop new and more effective therapeutic tools, as low level laser therapy did not demonstrate any additional benefits to ulcer healing with the parameters used in this study.

**Trial Registration:**

The trial was registered at ClinicalTrials.gov as NCT00860717.

## Background

Leprosy is a chronic infectious disease caused by *Mycobacterium leprae*, the only pathogenic bacteria able to infect peripheral nerves. About 30% of people with leprosy develop nerve damage. Neural impairment results in a set of sensitive, motor and autonomic disturbances, with ulcers originating primarily on the hands and feet. Neuropathic ulcers are one of the most common sequelae of leprosy, but little is known about their clinical and epidemiological aspects. They are very disabling to the patient and can result in deformity and/or amputation of the affected limb [1,2].

Brazil has the highest prevalence of leprosy cases in the world (3.21 cases per 10000 inhabitants in 2007), with the majority of these cases registered in the North and Middle-West Regions [3]. The State of Pará, in the Amazon region, registered 4955 new leprosy cases in 2006, accounting for nearly 1% of all cases world-wide [4].

Approximately 19% of new cases in Pará have a grade 1 or 2 disability. In 2005, the cure rate for leprosy was 71%, which was a questionable result according to Brazilian Health Ministry [5,6]. Delayed diagnostics, lack of appropriate treatment and failure in leprosy reactions control contribute to the occurrence of nerve damage and neuropathic ulcers in these patients.

Different methods of treatment have been used in ulcer management, but the outcomes are frequently dissatisfactory, and many people must live with chronic wounds that result in high economic and social costs [2].

Low level laser therapy (LLLT) has been used to accelerate wound healing since the late 1960 s, but its results are controversial [7]. One study [8] evaluated the use of LLLT in the treatment of leprosy ulcers with satisfactory results (66% were cured). However, in a systematic review published by Cochrane [9], the authors did not find evidence of wound healing improvement related to LLLT.

The main objectives of this study were to analyze clinical and epidemiological characteristics of patients with leprosy ulcers and to evaluate the effect of LLLT on wound healing in these patients.

## Methods

This study was approved by the Center of Tropical Medicine Research Ethics Committee from the Federal University of Pará (protocol number 074/2006 - CEP/NMT). The trial was registered at ClinicalTrials.gov as NCT00860717.

### Setting and Participants

The present study was done at the dressing service of Dr. Marcello Candia Reference Unit in Sanitary Dermatology of the State of Pará in Brazil (UREMC), and was carried out from January 2007 to January 2008. Participating subjects met the following inclusion criteria: (1) presented with neuropathic ulcer; (2) attended at least 3 weekly appointments at the dressing service of UREMC; (3) completed specific multi-drug therapy for *M. leprae*; and (4) gave written informed consent to participate in the study. There were no restrictions on gender, race or age-group, or the duration of ulcers. Subjects with the following conditions were not allowed to participate or were excluded from the study: (1) clinically detectable infection in the ulcer; (2) use of drugs, like corticosteroids that could interfere with the wound healing process; (3) use of special dressings like hydrocolloid, calcium alginate, activated carbon or any kind of therapeutic procedure different from that used routinely for both groups of study; (4) non-attendance to therapeutic program (six sequential times or nine intercalated); (5) pregnancy; and (6) discomfort during treatment procedure.

Clinical and epidemiological evaluation of patients with leprosy attended at the dressing service of UREMC were performed before the beginning of the randomized clinical trial. We conducted anamnesis, identification of the regions affected by the lesions (including a photographic register) and measurement of ulcer depth and surface area. The area was measured using UTHSCSA ImageTool 3.0 software (University of Texas Health Science Center, San Antonio, USA).

### Randomization and Interventions

Patients selection was performed after examination of all subjects who attended the dressing service from January to March, 2007. After initial assessment, subjects were randomly allocated into two groups of study, a control group (CG) and an experimental group (EG). Sample size was determined by the total number of patients that met the inclusion criteria and agreed to participate in this study. The randomization schedule was generated using BioEstat 5.0 software (Sociedade Civil Mamirauá, Amazonas, Brazil) after inclusion criteria had been evaluated and was done by random sampling. The subjects received a code related to the order in which they were evaluated. After all participants were recruited, they were allocated to the CG or the EG according to a sequence generated by the BioEstat 5.0 software. All stages of the randomization process were performed by the same researcher (JGB).

Subjects from the CG received routine treatment, including daily simple dressings with sterile gauze after wound cleaning with a 0.9% physiologic solution, use of 1% hydrophilic silver sulfadiazine cream (Prati Donaduzzi Laboratory, Paraná, Brazil) and orientation about the use of adapted footwear, self-care and the prevention of disabilities. Surgical debridement was done whenever indicated by nursing or orthopedic services from UREMC. Subjects from the EG received LLLT 3 times per week for 12 weeks, in addition to the same treatment as patients from the CG.

The LLLT equipment was a TWIN LASER (MM Optics, São Paulo, Brazil), an indium-gallium-aluminnium-phosphide (InGaAlP) semiconductor laser with a maximum output power of 40 mW, continuous radiation emission of visible red light with 660 nm wavelength (+/- 10 nm) and a spot area of 0.04 cm^2^. The energy density used was 4 J per point in the wound edges and 2 J/cm^2 ^in the wound bed with a power density of 1 W/cm^2^.

Wound beds were irradiated using a scanning technique with no direct contact. The laser probe was held upright to the ulcer during the treatment session and kept 1 cm away from the target tissue. Wound edges were treated using a "spot# technique, 1 cm from its border. Irradiated points on the wound edge were separated by approximately 1 cm. A contact technique was used in these sites by holding the laser probe upright to the ulcer edge. Direct skin contact was prevented by fixing a piece of transparent and disposable polyvinyl chloride (PVC) to the laser probe. The area of each ulcer was determined and the intended energy density for the time of the treatment session was calculated using the following equation: T = D × A/P, where (T) is time in seconds, (D) is energy density in J/cm^2^, (A) is bed ulcer area in cm^2 ^and (P) is the irradiance power in Watts.

All subjects included in this trial were evaluated biweekly until the end of the 12 weeks treatment period or until complete cicatrization of the treated ulcer. The last assessment was done one week after the last treatment session. The laser device used in this trial emitted red visible light and thus limited our ability to blind patients, as they could see the irradiation even when protective spectacles were used. In order to avoid duplicity in treatment technique and assessment interpretation, all LLLT and ulcer evaluation procedures were performed by one researcher (JGB).

### Outcomes

Ulcer area, depth and pressure ulcer scale for healing tool score (PUSH) were investigated in the clinical trial. Digital photographs were taken to evaluate ulcer area and analyzed using UTHSCSA ImageTool 3.0 software. To evaluate ulcer depth, a sterilized pincer was gently introduced into the bottom of the deepest region of the ulcer and measured from the tip to the surface of the skin. The PUSH tool score was submitted to a cross-cultural adaptation to the Portuguese language [10] and it resulted in the following sub-scores: surface area, exudate amount and type of wound tissue. This score ranged from 0 to 17, where 0 indicated a healed wound and 17 indicated an ulcer of more than 24 cm^2 ^of surface area, with a heavy amount of exudate and the presence of necrotic tissue.

### Statistical analysis

The collected data were submitted to descriptive analysis and methods of statistical inference using BioEstat 5.0 software. Statistical significance was assessed using a significance level of 0.05. The Student's *t *test was used to assess quantitative data of related samples, before and after treatment, and quantitative data of independent samples (CG and EG). The Wilcoxon test and the Mann-Whitney test were used to assess ordinal data of related and independent samples, respectively. The Fisher's exact test was used to assess the gender proportion and the use of adapted footwear between CG and EG, as well as the ulcer localization in different groups. The *Z *test was used to assess the surface area and depth of plantar ulcers compared to those on the legs or ankles.

## Results

### Clinical and epidemiological study

A total of 51 patients, with a mean age of 59.9 years old (minimum of 17 and maximum of 82), were evaluated in the early clinical and epidemiological study. Forty subjects (78%) were male and eleven (22%) were female. Leprosy was diagnosed an average of 27.6 years ago (SD= 18.7) and patients were cured of *M. leprae *infection 11.6 years ago (SD= 6.3). The most common clinical form was lepromatous leprosy, which corresponded to 72% of all cases.

**Figure 1:**
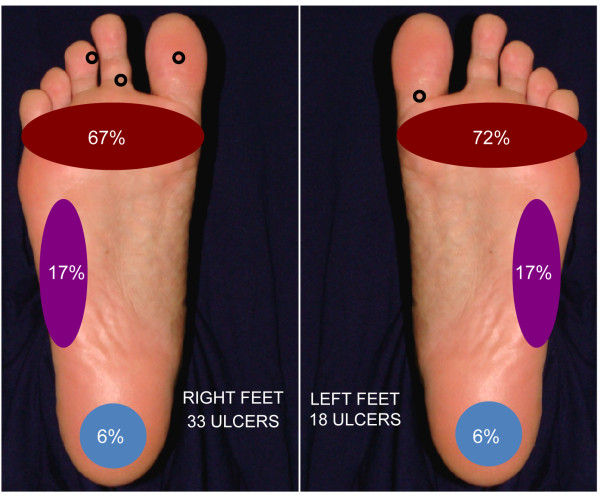
**Anatomic distribution of the ulcers on the plantar region**. Of 51 plantar ulcers, 33 were on right feet and 18 were on left feet. Ulcers were more common on the areas of the sole that bear weight during walking and standing. The little black circles indicate isolated lesions that were not included in the pictured percentages.

**Figure 2:**
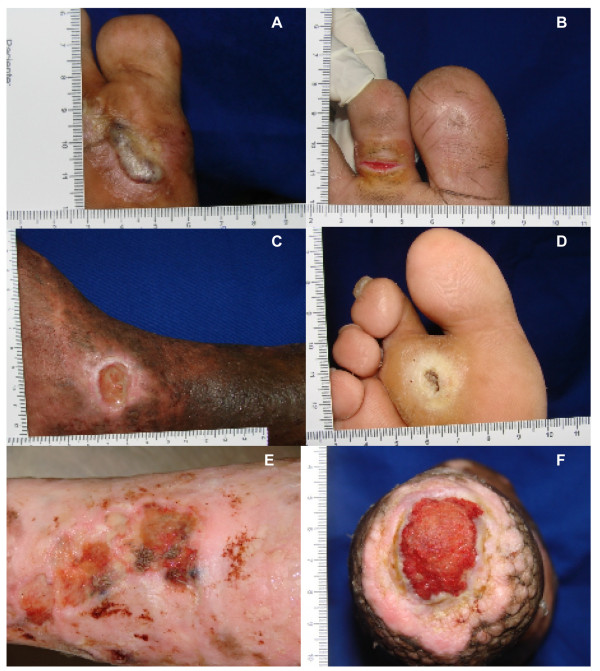
**Examples of ulcers in patients with leprosy**. **(A) **Blister formed after walking long distances that later became an ulcer. **(B) **Fissure on the base of the second toe of the right foot. **(C) **Right medial malleolus ulcer from a patient with leprosy and HBP. **(D) **Plantar ulcer on the region of second metatarsal head. **(E) **Myiasis in a chronic leg ulcer. **(F) **Chronic ulcer on a lower limb stump.

Ninety-seven ulcers were identified on the evaluated patients. On average, ulcer duration was 97.6 months (SD= 111.7), surface area was 7.3 cm^2 ^(SD= 11.5) and depth was 6.0 mm (SD= 6.2). The majority of the ulcers (53%) was located on the plantar region of the foot and were primarily distributed on those areas that bear weight while walking and standing, especially at the metatarsals head area, the fifth metatarsal bone and calcaneus area (Figure 1). Forty ulcers (41%) were located on legs and ankles and six lesions (6%) were located on hands and dorsal face of the feet. Different types of lesions were found, varying from blisters and fissures to extensive ulcers with secondary infestation, like myiasis (Figure 2). Besides, ten patients had a fibrotic skin surrounding the ulcers, four had fibrosis with lymphedema, and one presented only lymphedema. A striking difference was observed in area and depth between ulcers found on the plantar region (area: 4.2 cm^2^, depth: 7.2 mm) and ulcers on legs or ankles (area 13.2 cm^2^, depth: 4.0 mm) (Table 1).

**Table 1:** Ulcer area and depth according to location^a^.

Localization	Number of ulcers	Area (cm^2^)	Depth (mm)
Plantar	51	4.2 (6.2)	7.2 (7.5)

Legs/Ankles	40	13.2 (15.7)	4.0 (1.4)

Mean difference between groups (95% CI)		-9.23 (-9.35 to -9.11)	3.10 (3.06 to 3.15)

*p value*		<.001*	<.01*

^a^Values are means (SDs) unless otherwise indicated. CI = confidence interval

*As determined by the Z test for two independent samples.

There was a large number of lesions on the legs and ankles of patients, but they were especially prevalent among those who were more than 40 years old. The data indicate that ulcer location is inverted between patients less than 40 years old and those over 40. Only 18% of ulcers from younger patients were located on legs or ankles, while 46% of ulcers from patients over 40 years old were on the legs or ankles (Table 2).

**Table 2:** Ulcer occurrence and location by age-group and on patients with and without high blood pressure (HBP).

Subjects	Number of ulcers	Plantar	Legs and ankles	Other sites
≤ 40 years (n = 13)	17	13 (76%)	3 (18%)	1 (6%)

> 40 years (n = 38)	80	38 (48%)	37 (46%)	5 (6%)

*p *value^a^		.02	.02	.71

With HBP (n = 13)	40	14 (35%)	22 (55%)	4 (10%)

Without HBP (n = 38)	57	37 (65%)	18 (32%)	2 (3%)

*p *value^a^		<.01	.01	.18

^a^As determined by Fisher's exact test.

There were 13 cases (25%) of systemic high blood pressure (HBP), making it the most frequent comorbidity. The anatomic distribution of ulcers in patients with HBP assumed peculiar characteristics, as they located preferentially on the legs or ankles, when compared to subjects without HBP (Table 2).

Patients have an average of 3.4 simple dressings per week at the dressing service of UREMC, resulting in an estimated expenditure of $100.000 USD per year on disposable dressing material alone. Despite the availability of a complete orthopedic workshop that makes many kinds of adapted footwear that are freely distributed, 44% of evaluated patients did not use adapted footwear or an equivalent.

### Clinical trial

**Figure 3:**
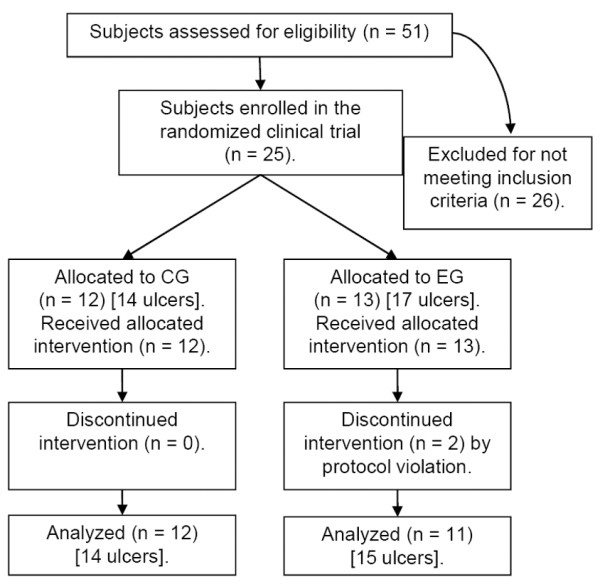
**Flow of participants through the trial**.

Of the 51 patients evaluated at the beginning of the trial, 25 were randomly allocated into the two trial groups (Figure 3). During the study follow-up, two subjects from the EG left the trial. One subject exceeded the protocol absence limit due to family problems and the other subject asked to drop out after 14 irregular sessions of LLLT, as he did not perceive any improvement in his ulcers. Subject demographics and baseline clinical characteristics that were included in the statistical analysis are described in Table 3. The data demonstrate that the CG and the EG were homogenous at the start of the clinical trial.

**Table 3:** Demographics and baseline characteristics by treatment group^1^.

Clinical data	Control group	Experimental group	*p *value
Gender (male/female)	9/3	9/2	1.0^a^

Age, years	58.5 (19.2)	53.3 (15.2)	.42^b^

Most common clinical form (%)	LL^# ^(92%)	LL^# ^(73%)	.31^a^

Years since leprosy diagnostic	38.4 (16.0)	39.1 (15.7)	.91^b^

Years free of *M. leprae*	14.2 (5.6)	14.3 (3.9)	.97^b^

Ulcers duration (months)	71.7 (82.2)	123.3 (159.6)	.28^b^

Ulcers area	5.3 (9.2)	4.2 (5.9)	.70^b^

Ulcers depth	6.3 (5.4)	6.2 (5.1)	.96^b^

PUSH score	9.7 (3.4)	9.6 (3.3)	.96^c^

Adapted footwear usage	5 subjects	4 subjects	1.0^a^

^1^Values are means (SDs) unless otherwise indicated. ^# ^LL = Lepromatous leprosy.

^a ^As determined by Fisher's exact test.

^b^As determined by an independent 2-sample *t *test.

^c^As determined by the Mann-Whitney *U *test.

The surface areas, depths and PUSH scores of the ulcers of patients in both study groups, both before and after the treatment period, are recorded in Table 4. The data indicate that there were no statistical differences in any of the investigated variables, including the subscores for exudate amount and type of wound tissue. No treatment-related adverse effects were reported during this study.

**Table 4:** Results of Analysis Comparing Outcomes: Evaluation Within Groups and Between Treatment Groups.^a^

Outcome measure	Control group (n = 12)	Experimental group (n = 11)	Mean difference between groups (95% CI)	*p *value^d^
**Area (cm^2^)**				

Baseline	5.3 (9.2)	4.2 (5.9)	-1.08 (-6.93 to 4.75)	.70

After intervention	4.4 (8.5)	3.8 (5.7)	-0.63 (-6.10 to 4.84)	.81

Mean difference in change scores (95% CI)	0.82 (0.00 to 1.66)	0.37 (-0.77 to 1.51)		

*p *value^c^	.05	.49		

**Depth (mm)**				

Baseline	6.3 (5.4)	6.2 (5.1)	-0.08 (-4.07 to 3.90)	.96

After intervention	5.4 (5.7)	4.1 (3.9)	-1.29 (-5.00 to 2.41)	.47

Mean difference in change scores (95% CI)	0.85 (-0.40 to 2.11)	2.0 (-0.60 to 4.74)		

*p *value^c^	.16	.11		

**PUSH score^b^**				

Baseline	9.7 (3.4)	9.6 (3.3)		.96^e^

After intervention	8.4 (5.3)	7.9 (5.3)		.93^e^

*p *value^e^	.09	.24		

^a^Values are means (SDs) unless otherwise indicated. CI = confidence interval.

^b ^PUSH = Pressure Ulcer Scale for Healing (ranged from 0 to 17, where 0 indicated a healed wound and 17 indicated an ulcer greater than 24 cm^2 ^in surface area, with a heavy amount of exudate and the presence of necrotic tissue).

^c^As determined by a dependent sample *t *test.

^d^As determined by an independent 2-sample *t *test.

^e^As determined by the Mann-Whitney *U *test.

## Discussion

Ulcers in patients with leprosy can remain for several years after the initial infection is resolved and can result in large economic and social losses. Such losses were observed in this study, which was primarily composed of former patients that have lived with their ulcers for many years. The most important causal factor for neuropathic foot ulcers is the presence of a dynamic or static deformity leading to local areas of peak pressure on insensitive skin, which has been well illustrated by pressure studies [11]. This repetitive overload on specific areas of the sole could partially explain why plantar ulcers are deeper and smaller than leg and ankle ulcers. Almost half of the evaluated subjects in this study did not use any kind of adapted footwear, suggesting some negligence by the patients in the prevention of disabilities and self-care procedures. The free distribution of special footwear doesn't ensure its adequate utilization. Health care workers need to be constantly pushed to establish a patient continuum education process about self-care routines and to improve the techniques currently employed to encourage the use of preventive tools. Low adherence to such programs and self-care procedures is a concern of countries that still bear a significant leprosy burden.

In the present study, patient ulcers were predominantly chronic wounds, which could have interfered with LLLT success. Fibroblasts in chronic wounds have impaired responsiveness to growth hormone, which may be due to an increased number of senescent cells [12]. It was observed in neuropathic diabetic foot ulcers that wound duration negatively affected the chance of healing after 12 weeks of proper wound care [13]. A high prevalence of vasomotor reflex impairment attributed to autonomic nerve lesions has been observed in newly diagnosed patients with leprosy [14]. Lepromatous patients exhibit a tendency to develop chronic leg ulcers, which are partially caused by a single vascular disturbance during *M. leprae *infection and/or by peripheral neuropathy [15]. These alterations may explain the notable occurrence of chronic leg and ankle ulcers in this sample, especially in subjects who were hypertensive or over 40 years old. One study of 124 patients with leg wounds of different etiologies identified that 54% of them were hypertensive [16]. It is necessary to give special attention to the control of high blood pressure, as this comorbidity was common in our study and has the potential to negatively affect the wound healing process in patients with leprosy. The follow-up period of 12 weeks is longer than some previous studies [8,17-19], but was chosen because it is enough time for the wound healing process to complete [20-22].

Rest is a common recommendation as a strategy of self-care, but it was not adopted by the majority of patients. Such behavior combined with the non-use of adapted footwear (though use was advised by the health care staff) could have interfered with the results, as the plantar ulcers remained under mechanic stress during daily living activities and walking. The fact that researchers were not blind in the study and therefore knew which group subjects were receiving laser therapy or routine treatment could have led to a possible bias, but the photographic register of all treated ulcers allows confirmation of collected and analyzed data.

There was no formally sample size calculation, but we included all subjects attended at dressing service of UREMC that met the inclusion criteria. The small sample size of this clinical trial limits the application of the data in other settings or studies, and does not provide robust evidence of no effect of laser in these wounds. More studies with larger sample sizes are necessary and should include different research institutes and universities, as well as additional control over self-care and prevention of disabilities procedures.

Even though the supporting evidence is weak, LLLT has been used by health care professionals in many countries around the world for the treatment of venous, pressure and diabetic chronic wounds [23]. Our results disagree with those obtained by one previous study [8], where a wound cure rate of 66% was reached. However, that study was greatly limited as the authors included only four patients (12 ulcers) and had no control group. Many *in vitro, in vivo *and human studies report positive effects of LLLT [24-33], though there are other works that did not reach the same conclusion [34-41]. One systematic review of papers published after 1999 [42] concluded that there is no sufficient scientific evidence to support the use of LLLT for wound healing. The author declares that new controlled studies are necessary to determine its real efficacy and to delimitate more adequate procedures for each group of patients.

Although their focus was on a different primary disease, our results are in accordance with a systematic review by Flemming and Cullum published by Cochrane Library [9]. They found no evidence that treatment with LLLT could provide any benefit for venous leg ulcer healing. However, in one meta-analysis [43], the authors concluded that LLLT is an effective tool to promote wound healing. These conflicting results may be partially attributed to disparities in study design, including different laser types, variance of treatment parameters and selected samples. The evaluated papers reported no side effects related to exposure to LLLT.

Management of chronic ulcers in patients with leprosy includes different types of dressings, orthopedic and plastic surgeries, plaster casts, special footwear, splints, crutches, wheelchair use and absolute rest. Despite this, clinical experience shows that patient compliance to the therapeutic procedures is a key consideration in treatment choice and that without patient collaboration the result of the treatment can be frustrating. Low patient adherence to rehabilitation and prevention of disabilities programs (e.g. usage of appropriate footwear) indicate that more research and educational measures are necessary to improve the adoption of such strategies. More research is also needed to develop more efficient therapeutic tools.

## Conclusions

Ulcers in patients with leprosy sequelae remain a major source of economic and social losses, even many years after they have been cured of *M. leprae *infection. With the parameters used in this study, low level laser therapy did not demonstrate any additional benefit to ulcer healing for these patients, when compared to patients in the control group.

## Competing interests

The authors declare that they have no competing interests.

## Authors' contributions

JGB and CGS designed the study; JGB performed data collection; JGB and CGS analyzed the data, wrote the first draft of the manuscript, read and approved the final version of the manuscript.

## Pre-publication history

The pre-publication history for this paper can be accessed here:

http://www.biomedcentral.com/1471-2334/10/237/prepub
